# Chinese Tallow Trees (*Triadica sebifera*) from the Invasive Range Outperform Those from the Native Range with an Active Soil Community or Phosphorus Fertilization

**DOI:** 10.1371/journal.pone.0074233

**Published:** 2013-09-02

**Authors:** Ling Zhang, Yaojun Zhang, Hong Wang, Jianwen Zou, Evan Siemann

**Affiliations:** 1 College of Resources & Environmental Sciences, Nanjing Agricultural University, Nanjing, China; 2 Department of Ecology & Evolutionary Biology, Rice University, Houston, Texas, United States of America; Helmholtz Centre for Environmental Research - UFZ, Germany

## Abstract

Two mechanisms that have been proposed to explain success of invasive plants are unusual biotic interactions, such as enemy release or enhanced mutualisms, and increased resource availability. However, while these mechanisms are usually considered separately, both may be involved in successful invasions. Biotic interactions may be positive or negative and may interact with nutritional resources in determining invasion success. In addition, the effects of different nutrients on invasions may vary. Finally, genetic variation in traits between populations located in introduced versus native ranges may be important for biotic interactions and/or resource use. Here, we investigated the roles of soil biota, resource availability, and plant genetic variation using seedlings of *Triadica sebifera* in an experiment in the native range (China). We manipulated nitrogen (control or 4 g/m^2^), phosphorus (control or 0.5 g/m^2^), soil biota (untreated or sterilized field soil), and plant origin (4 populations from the invasive range, 4 populations from the native range) in a full factorial experiment. Phosphorus addition increased root, stem, and leaf masses. Leaf mass and height growth depended on population origin and soil sterilization. Invasive populations had higher leaf mass and growth rates than native populations did in fresh soil but they had lower, comparable leaf mass and growth rates in sterilized soil. Invasive populations had higher growth rates with phosphorus addition but native ones did not. Soil sterilization decreased specific leaf area in both native and exotic populations. Negative effects of soil sterilization suggest that soil pathogens may not be as important as soil mutualists for *T. sebifera* performance. Moreover, interactive effects of sterilization and origin suggest that invasive *T. sebifera* may have evolved more beneficial relationships with the soil biota. Overall, seedlings from the invasive range outperformed those from the native range, however, an absence of soil biota or low phosphorus removed this advantage.

## Introduction

Exotic plant invasions threaten ecosystem functions and stability [1]–[3]. Identifying the mechanisms underlying successful plant invasions will help guide effective invasive plant control and aid in ecosystem restoration. Two mechanisms that have been proposed to explain successful plant invasions are: 1) that exotic plants benefit from greater resource availability (the increased resource availability hypothesis or “IRAH”; [4], [5]) and 2) exotic plants benefit from weak effects of natural enemies (the enemy release hypothesis or “ERH”; [6]) and/or strong effects of mutualists (the enhanced mutualists hypothesis or EMH, [7]).

The IRAH posits that the opportunities for invasions increase as resource availability increases in a community [4]. This increased resource availability does not necessarily reflect higher nutrient input because resource availability reflects the balance of resource supply and uptake by resident plants [5]. While most, but not all, exotic invaders may be better adapted to high nutrient conditions than native species (“ruderals” [8]), pre-adaptation or post-introduction adaptation of exotic plants to high nutrient conditions may confer an advantage to exotic plants compared to less well-adapted native plants. For instance, invasive plants may be favored by increased soil resources (*e.g.* nitrogen [N], phosphorus [P]) that favor plants with low root to shoot ratios [9]. Similarly, plants with high N dependent maximal growth rates will be favored over those with high N use efficiencies when that N availability is high. Because plants with high N demand may not also have high P demand, for instance because of different symbiotic relationships (*e.g.* rhizobial or mycorrhizal) or allocation to high N (proteins) or P (nucleic acids) compounds, soil resources may vary in their impacts on invasions [2], [10]. Moreover, nutrient assimilation by invasive plant species may vary due to positive and/or negative biotic interactions with more positive or less negative interactions facilitating nutrient uptake of the host plant.

The ERH posits that exotic plants benefit from introduction to a new range without specialist enemies in combination with not being preferred by generalist enemies [6]. Recent studies suggested that escape from soil pathogens may be at least as important as escape from aboveground specialist insect herbivores in their contribution to successful plant invasions [11]–[13]. Since soil communities include pathogens, parasites, and herbivores as well as beneficial groups (*e.g*. mycorrhizae, rhizobia) [14], [15], the overall impact of soil biota on plant performance will reflect the net effect of both negative and positive interactions [16]. Strong negative impacts of soil microbial communities on invasive plants have mostly been observed in natural population of these plants growing in their native ranges [11], [17] indicating that negative interactions are relatively stronger than beneficial ones [16]. This could reflect stronger negative effects or weaker positive effects on plant performance [7], [18], [19].

Differences in biotic or abiotic factors between the native and invasive ranges of plants can lead to genetic differences in morphological or physiological traits between populations in the native and introduced ranges [20]–[23]. One example of a shift in morphological traits is a lower root to shoot ratio [24], [25]. In general, a lower root to shoot ratio provides an advantage in competition for aboveground resources and a disadvantage in competition for belowground resources [9]. In addition, escape from natural enemies, in particular specialists, in the invasive range may lead to a reallocation from defense to growth [26]–[28]. Moreover, more beneficial soil mutualisms in the invasive range [11] may lead to genetic differences in plant traits relevant to these interactions. However, resource requirements and biotic interactions are not independent [29], [30]. In addition, shifts in traits of invasive plants may lead to altered soil microbial communities [25], [31], which may in turn impact soil N and P use [32]–[35]. However, the dependence of invasive plant performance on genetic variation in plant traits, interactions with the soil biota, and availability of N and P is poorly understood.

Here, we examined effects of interactions between soil nutrients (N and P), soil microbial communities (active or sterilized), and population origin (native or invasive range) using Chinese tallow tree (*Triadica sebifera* (L.) Small, henceforth *T. sebifera*) as a model plant. *T. sebifera* is native to China and was first introduced into the USA in 1772 to Savannah, GA then subsequently to several sites along the Gulf Coast and is now invasive in grasslands, forests, and disturbed habitats throughout the southeastern USA, converting them to monospecific forests [36]–[39]. Previous studies have demonstrated that invasive *T. sebifera* had unusually positive interactions with the soil biota relative to native tree species in the introduced range [40]. Conducting studies in the native range with populations from the native and introduced ranges provides additional insights into how genetic differences in *T. sebifera* populations may influence the net effects of the soil biota on *T. sebifera* performance. In an experiment conducted in the native range, we addressed the following questions: (1) Do *T. sebifera* seedlings perform better with N and/or P addition? (2) What are the net effects of the soil biota in the native range? (3) Do *T. sebifera* seedling responses to nutrient additions and soil biota manipulations differ between population origins?

## Materials and Methods

### Focal Species

*T. sebifera* is native to China where it has been cultivated for 14 centuries and is now an aggressive invader in the southeastern USA [36], [41]. Studies demonstrated *T. sebifera* in the invasive range (invasive populations) are faster-growing relative to native conspecifics (native populations) or non-invasive co-occurring plant species [25], [27], [42]. Invasive *T. sebifera* rapidly accumulates soil pathogens in the invasive range relative to co-occurring native resident species which decreases the performance of *T. sebifera* seedlings under conspecifics [43], [44]. However, *T. sebifera* has also been shown to be more mycorrhizal dependent in its invasive range compared with native trees [40], [44]. In addition, *T. sebifera* seedlings from the invasive range have stronger responses to N addition than ones from the native range perhaps partly due to facilitation of N mineralization [34].

### Seeds and Seedlings

In November 2009, we hand collected seeds of naturalized *T. sebifera* in China and the USA (Table 1). All seed collections were from public areas where no permission was required for collection. *T. sebifera* is not an endangered or protected species in either country. All seeds were collected from at least five haphazardly selected trees. Seeds used for planting were weighed by populations to evaluate the potential impacts of seed provisioning on seedling performance. Results of an ANOVA showed that seed masses of populations were independent of population origin (*F_1,6_* = 3.99, *P = *0.09). Also, seedling height (*F_1,6_* = 0.25, *P* = 0.64) and number of leaves (*F_1,6_* = 2.59, *P* = 0.16) at the time of transplanting were independent of population origin. Together these results suggest that there were no strong maternal effects due to differences in seed provisioning. In January, we treated seeds in a 10% bleach rinse and then soaked seeds in water with lab detergent to remove the waxy seed coat [43]. All seeds were then surface sterilized by 0.5% potassium permanganate and planted in 100 ml Conetainers (Stuewe & Sons, Corvallis, OR, USA) filled with sterilized field soil (see below). Seeds germinated in early April, 2010. After seedlings had secondary leaves, seedlings of similar heights were transplanted into pots (1.5 L). Pots received three soil treatments in a full-factorial design (N = 256, 2 population origins×4 populations×2 soil sterilization×2 N×2 P×4 replicates). To coincide with the growing season of *T. sebifera* in this area seeds were grown for 4 months in a non-heated greenhouse from June 2010 to November 2010 at Nanjing Agricultural University, Nanjing, China.

**Table 1:** Native (China) and invasive (USA) *T. sebifera* populations used in this experiment.

Source population	Latitude	Longitude
**China**		
Hefei, Anhui	31°38∼39′N	117°50∼51′E
Bengbu, Anhui	32°57∼58′N	117°20∼21′E
Nanjing, Jiangsu	32°02∼03′N	118°50∼51′E
Shanghai	31°31∼32′N	121°52∼53′E
**USA**		
Limehouse, SC	32°09∼10′N	81°05∼07′W
Hutchinson Island, GA	31°23∼24′N	81°15∼16′W
Houston, TX	29°41∼42′N	95°25∼26′W
Gainesville, FL	29°34∼35′N	82°21∼22′W

### Soil Treatments

Soil was collected from the top 20 cm in a fallow agricultural field. *T. sebifera* trees were at least 200 m away from where soil was collected to reduce the potential buildup of specific soil organisms [16]. Soils characteristics were: carbon % = 2.32±0.11; nitrogen % = 0.22±0.007; C:N = 10.53±1.65 (means ±1 se). Previous studies focused on home- and away-soil effects indicate buildup of negative soil organisms in conspecific (home) soils in both the native and introduced ranges [43], [44]. The soils used here are suited for making inferences about the effects of soil nutrients and the soil biota during the process of colonization in the native range and spread in the introduced range. Half of the soil was autoclaved at 121°C for 40 minutes (“sterilized soil”) and the other half was left untreated (“fresh soil”).

Pots that were in the N fertilizer treatment received 4 g m^−2^ of N as KNO_3_ (equivalent to 15.1 mg/L of soil). Plants in the control (no addition) N treatment received an equivalent volume of deionized water. Pots in the P fertilizer treatments received P at a rate of 0.5 g m^−2^ as KH_2_PO_4_ (equivalent to 1.9 mg/L of soil) and control (no addition) P pots received an equivalent volume of deionized water. Fertilizer additions were made one month after seedlings were transplanted.

### Data Collection

We measured stem height of each seedling from ground surface to terminal bud at both the beginning and the end of the experiment. We thoroughly cleaned equipment between measurements. At the end of the experiment (4 months), seedlings were clipped at ground level (then separated into leaves and stems) and roots were gently washed from the soil. Total leaf area (cm^2^) was obtained by scanning fresh leaves and analyzing them with SCNIMAGE (Scion Corporation; www.scioncorp.com). Seedling roots, stems, and leaves were then dried at 60°C to constant mass and weighed. We calculated height growth rates (HGR, mm cm^−1^ day^−1^) as: HGR = *ln* (harvest stem height/initial stem height at transplanting)/days. Specific leaf area (SLA, leaf area per unit leaf dry mass, cm^2^ g^−1^) was calculated dividing leaf area by leaf biomass.

### Statistical Analyses

We first conducted a MANOVA to examine the effects of seedling origin, N treatment, P treatment, soil treatment, and their interactions on *T. sebifera* root mass, stem mass, and leaf mass. We used variation among populations to test for differences between population origins (and corresponding interaction terms with population to examine interactive effects with origin). Because there were significant MANOVA results, we then conducted ANOVAs for each of the biomass variables. We also conducted ANOVAs to examine the dependence of height growth rate and specific leaf area on our treatments. We used partial difference adjusted means contrast tests to examine differences among treatment means for significant interactive effects. Data did not need to be transformed to meet the assumptions of ANOVA. Differences at α = 0.05 level are reported as significant. All statistical analyses were carried out in SAS (SAS Institute, Cary, NC, USA).

## Results

### Plant Biomass

**Figure 1:**
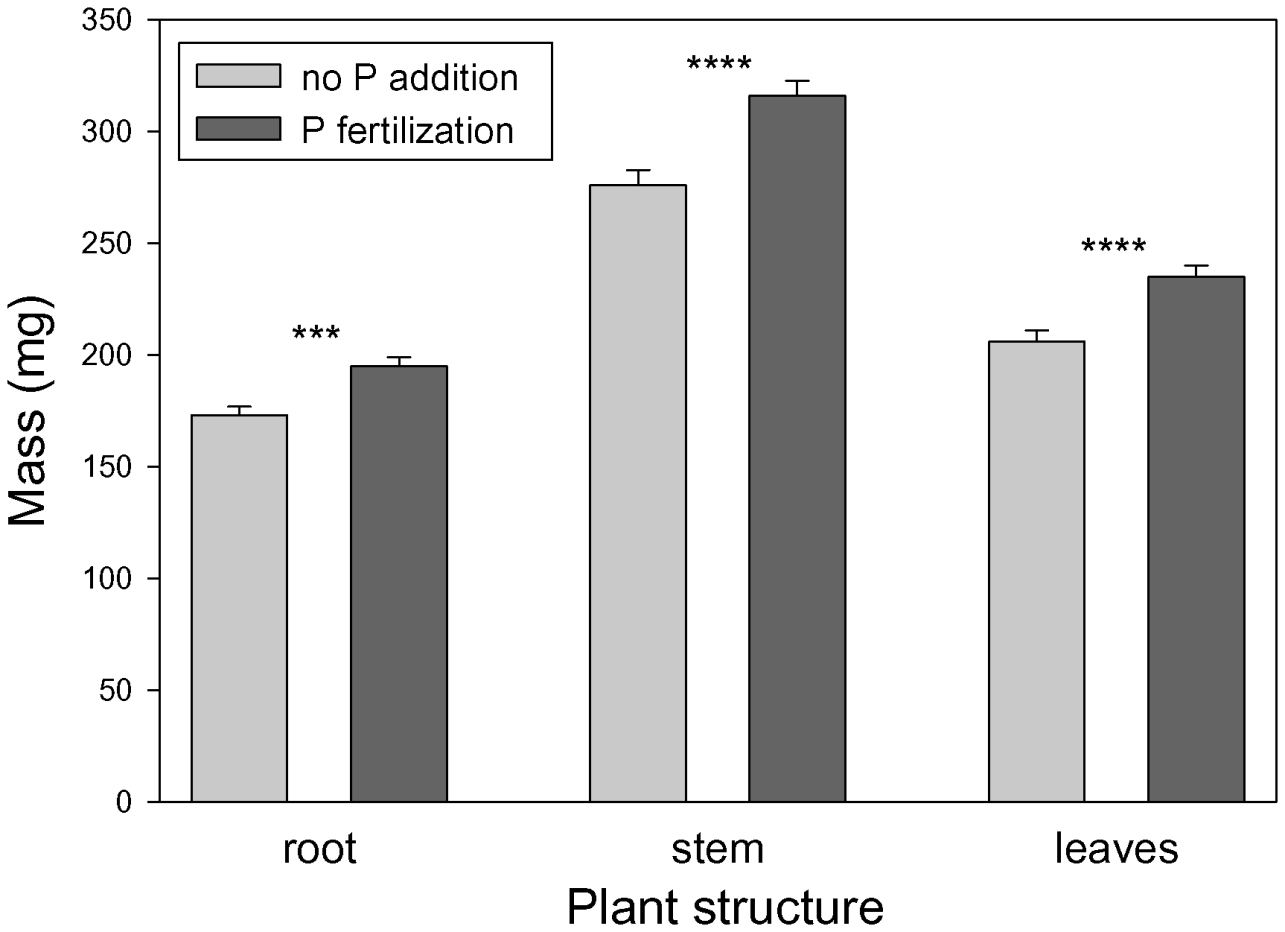
The dependence of root, stem and leaf masses of *T. sebifera* seedlings on P addition. Means+1 SE. ***: *P*<0.001; ****: *P*<0.0001.

**Figure 2:**
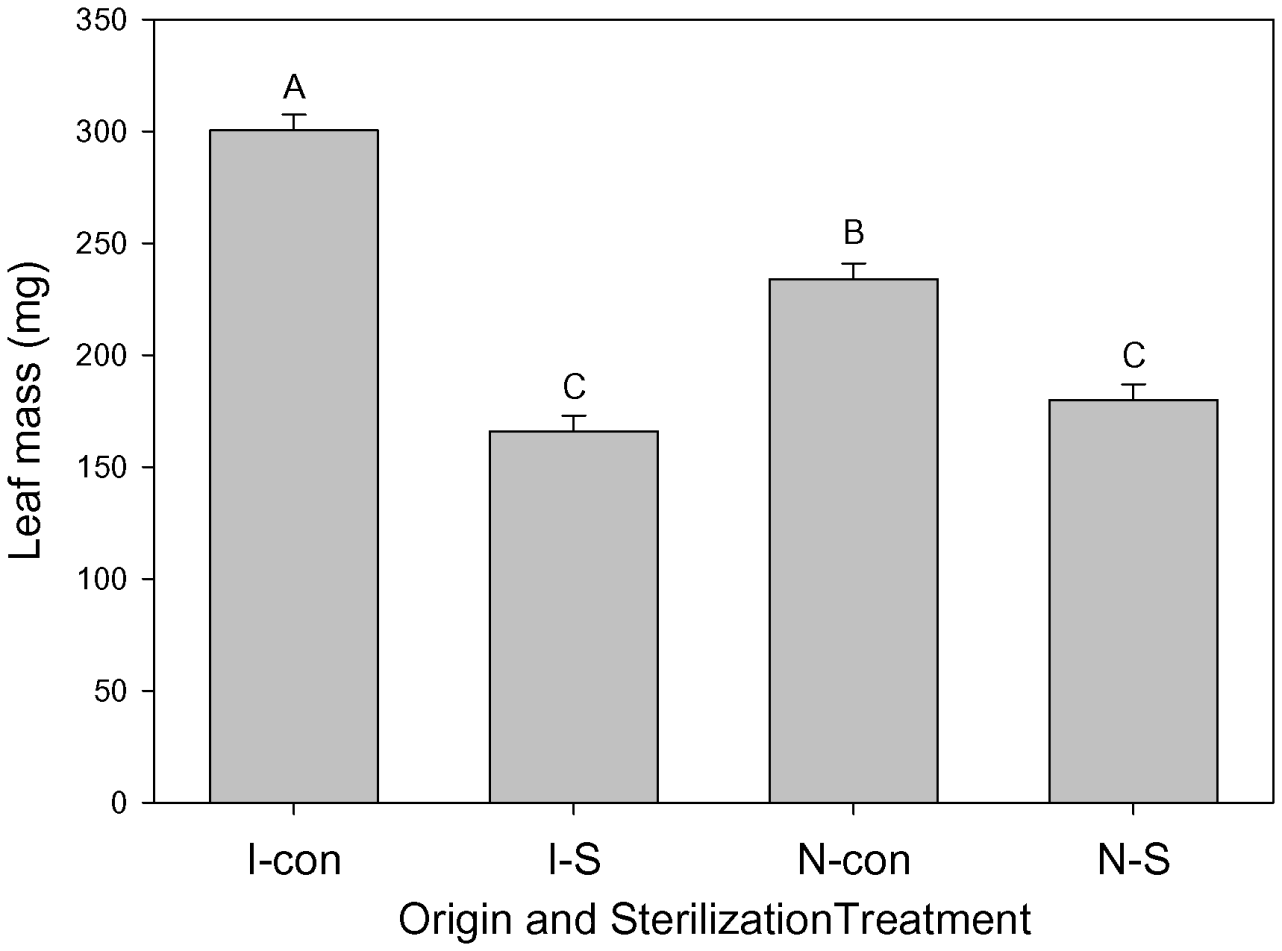
The dependence of leaf mass on population origin (“I” is invasive, “N” is native) and soil sterilization treatments (“con” is control, “S” is sterilization). Means+1 SE. Means with the same letter were not significantly different in post-hoc tests (P<0.05).

In the MANOVA, P addition, sterilization, origin×sterilization, and origin×N×P×sterilization all had significant effects on root, stem and leaf biomass (Table 2). In follow-up ANOVAs, P addition significantly increased biomass of roots, stems and leaves (Table 2; Fig. 1). In addition, leaf biomass depended on sterilization and origin×sterilization with greater increases in leaf biomass in fresh soil compared to sterilized soil for seedlings from invasive populations versus native populations (Fig. 2).

**Table 2:** The dependence of root, stem, leaf biomass on origin, N addition, P addition and soil sterilization and their interactions in a MANOVA and follow-up ANOVAs.

	MANOVA			Root			Stem			Leaf		
Effect	DF	F	P	DF	F	P	DF	F	P	DF	F	P
Origin	3,4	2.52	0.1971	1,6	1.09	0.3366	1,6	0.12	0.7446	1,6	5.35	0.0600
N	3,189	1.01	0.3911	1,191	2.40	0.1227	1,191	0.25	0.6167	1,191	0.18	0.6723
P	**3,189**	**7.15**	**<0.0001**	**1,191**	**12.20**	**0.0006**	**1,191**	**16.79**	**<0.0001**	**1,191**	**16.21**	**<0.0001**
Sterilization	**3,189**	**83.84**	**<0.0001**	1,191	0.23	0.6339	1,191	0.02	0.8918	**1,191**	**171.29**	**<0.0001**
Origin×N	3,4	0.48	0.7147	1,6	0.44	0.5323	1,6	0.42	0.5402	1,6	0.01	0.9915
Origin×P	3,4	3.95	0.1087	1,6	1.46	0.2717	1,6	0.09	0.7773	1,6	0.99	0.3589
Origin×Sterilization	**3,4**	**7.16**	**0.0437**	1,6	0.69	0.4376	1,6	0.48	0.5142	**1,6**	**14.52**	**0.0089**
N×P	3,189	0.59	0.6228	1,191	0.06	0.8117	1,191	0.25	0.6205	1,191	0.53	0.4684
N×Sterilization	3,189	1.75	0.1574	1,191	0.07	0.7855	1,191	0.32	0.5716	1,191	2.26	0.1348
P×Sterilization	3,189	1.26	0.2910	1,191	2.30	0.1311	1,191	3.56	0.0609	1,191	0.57	0.4509
Origin×N×P	3,4	0.22	0.8789	1,6	0.43	0.5385	1,6	0.42	0.5420	1,6	0.80	0.4056
Origin×N×Sterilization	3,4	0.39	0.7676	1,6	1.34	0.2914	1,6	0.88	0.3850	1,6	0.05	0.8314
Origin×P×Sterilization	3,4	0.81	0.5514	1,6	0.15	0.7111	1,6	0.06	0.8211	1,6	1.36	0.2872
N×P×Sterilization	3,189	0.57	0.6342	1,191	0.64	0.4243	1,191	0.01	0.9176	1,191	0.59	0.4445
Origin×N×P×Sterilization	**3,4**	**8.45**	**0.0332**	1,6	0.43	0.5385	1,6	1.39	0.2834	1,6	3.85	0.0973

Significant results shown in bold.

### Plant Growth Rate and Specific Leaf Area

**Figure 3:**
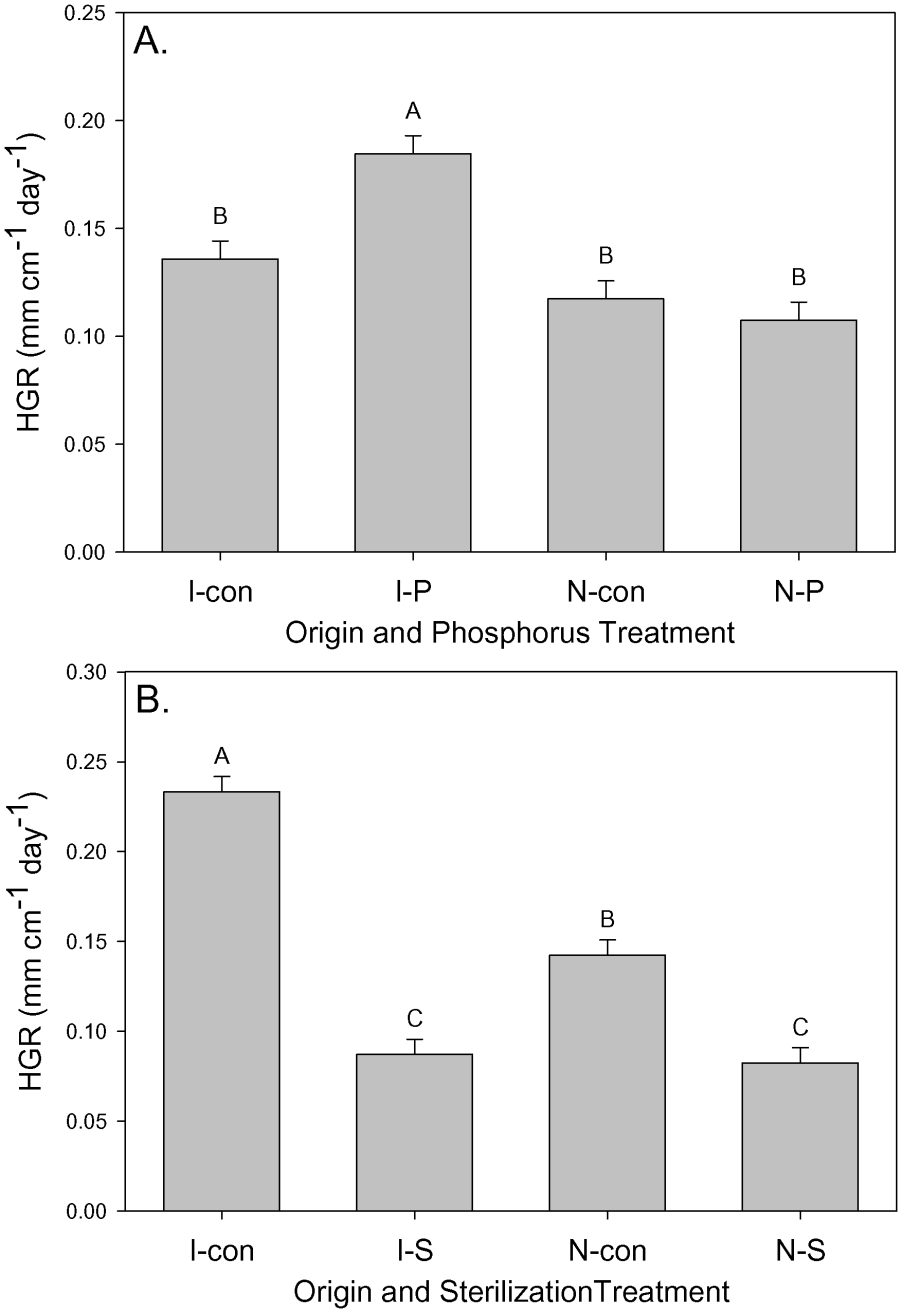
The dependence of height growth (HGR) on A) population origin (“I” is invasive, “N” is native) and P treatment (“con” is control, “P” is addition) and B) population origin and soil sterilization treatments (“con” is control, “S” is sterilization). Means+1 SE. Means with the same letter were not significantly different in post-hoc tests (P<0.05).

Height growth rate depended on origin, P addition, soil sterilization, origin×P, and origin×sterilization (Table 3). Seedlings from invasive populations had significantly higher growth rates with P addition but ones from native populations did not (Fig. 3A).

**Table 3:** The dependence of height growth rate (HGR) and specific leaf area (SLA) on origin, N addition, P addition and soil sterilization and their interactions in ANOVAs.

		HGR		SLA	
Effect	DF	F	P	F	P
Origin	1,6	**33.78**	**0.0011**	3.70	0.1026
N	1,233	0.02	0.8999	**5.67**	**0.0181**
P	1,233	**5.94**	**0.0156**	**4.65**	**0.0322**
Sterilization	1,233	**146.88**	**<0.0001**	**150.86**	**<0.0001**
Origin×N	1,6	2.91	0.1388	0.52	0.4979
Origin×P	1,6	**12.36**	**0.0126**	0.74	0.4218
Origin×Sterilization	1,6	**27.72**	**0.0019**	2.44	0.1691
N×P	1,233	0.34	0.5618	0.02	0.8884
N×Sterilization	1,233	1.11	0.2940	1.05	0.3074
P×Sterilization	1,233	0.82	0.3664	1.55	0.2145
Origin×N×P	1,6	0.09	0.7728	**8.51**	**0.0267**
Origin×N×Sterilization	1,6	0.35	0.5749	1.01	0.3528
Origin×P×Sterilization	1,6	1.03	0.3494	0.02	0.8811
N×P×Sterilization	1,233	0.18	0.6687	3.03	0.0832
Origin×N×P×Sterilization	1,6	0.04	0.8530	0.01	0.9739

Significant results shown in bold.

**Figure 4:**
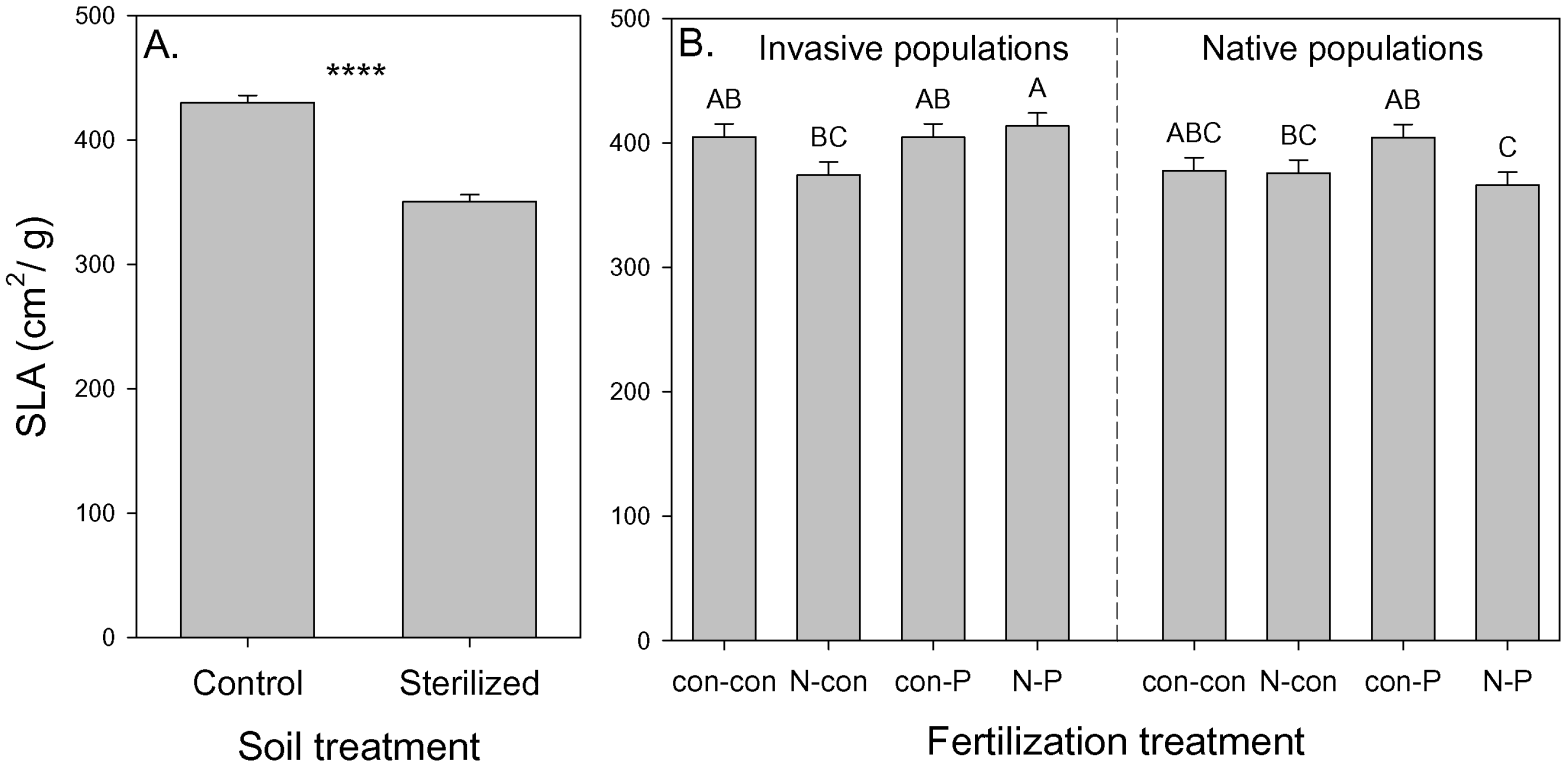
The dependence of specific leaf area (SLA) on A) soil treatment and B) population origin (“I” is invasive, “N” is native) and fertilization treatment (“con-con” is no fertilization, “N-con” is N addition, “con-P” is P addition, and “N-P” is N and P addition). Means+1 SE. Means with the same letter were not significantly different in post-hoc tests (P<0.05). ****: *P*<0.0001.

In addition, the height growth increases in fresh soil compared to sterilized soil were significantly larger for invasive populations (Fig. 3B). Specific leaf area was significantly higher in fresh soil (Fig. 4A) and SLA also depended on N addition, P addition, and origin×N×P (Table 3). This interactive effect reflected significantly higher SLA for seedlings from invasive populations but significantly lower SLA for those from native populations when both N and P were added (Fig. 4B).

## Discussion

Root, stem and leaf biomass of both origins were increased with P addition. In previous studies of plant invasions and soil P, most reported increased P availability in invaded areas [32], [33], [45]–[47] suggesting that invasive species may have evolved to mineralize soil P at a higher efficiency relative to native ones. Additional studies have demonstrated the importance of P availability for competitive ability and range expansion for invasive plant species [48], [49]. Our results indicated that seedlings from both native and invasive origins were P limited since each responded positively to P addition, but had no response to N addition. However, N is another important soil nutrient that may limit plant growth and range expansion of *T. sebifera*. Zou *et al.* [34] found higher soil organic N mineralization in soils associated with *T. sebifera* of invasive origin, which might lead to increased soil N availability. In addition, invasive *T. sebifera* plants have been shown to have a stronger positive response to inorganic N levels relative to those from native populations [34]. However, growth of *T. sebifera* seedlings from invasive populations invading coastal prairies in the introduced range responded significantly to N and K addition alone but only responded positively to P addition when N was also added [48]. The strong positive response to P addition but not N addition we found here may reflect the extremely high levels of N deposition in the native range of *T. sebifera* [50].

The negative effects of soil sterilization on leaf biomass and height growth rate suggested *T. sebifera* seedlings had net positive interactions with the soil biota in the native range. Specific leaf area also decreased with soil sterilization (Table 3; Fig. 4A). Higher SLA is usually associated with lower leaf construction cost and higher N use efficiency in invasive plants [51]–[53]. One interaction that is important for P assimilation by plant species is arbuscular mycorrhizal fungi [54]. The higher arbuscular mycorrhizal colonization level observed for invasive *T. sebifera* relative to the native tree species in the introduced range is evidence that *T. sebifera* is arbuscular mycorrhizae dependent [40]. In our study, soil sterilization interacted with seedling origin to impact leaf biomass, with invasive origin seedlings more strongly inhibited by soil sterilization relative to ones of native origin. Thus, it appears that *T. sebifera* from both origins have overall positive interactions with the soil microbial communities but that those interactions are more beneficial for those of invasive origin relative to those of native origin. Although our populations spanned a broad geographical range and included descendants of both introductions, including a larger number of populations may have increased the number of population origin effects that were significant.

Assuming the negative effect of soil sterilization was simply the removal of mutualists important in P or N uptake [10], [55], the negative effect of soil sterilization on growth might be weakened when N and/or P were added. However, there was not such a significant interactive effect on the mass of leaves, stems, or roots or on height growth rate. Perhaps uptake was so poor in sterilized soils that additional nutrients were not available to plants. The greater decline in leaf biomass for invasive origin plants relative those of native origin indicated a greater net beneficial interaction with the soil biota [40]. This could reflect greater positive interactions or weaker negative interactions but these possibilities cannot be evaluated in this study. If a similar pattern exists in the introduced range, it might be a mechanism contributing to its successful invasion.

Height growth rate of seedlings from the invasive range significantly increased with P addition but those from the native range did not respond to P addition (Table 3; Fig. 3). Generally, in a high resource, low stress environment, plants with a higher growth rate would be more successful when competing for light [56], [57]. There was a significant interactive effect of origin, N addition, and P addition in which seedlings from invasive populations had especially high SLA and seedlings from native populations had especially low SLA (Fig. 4B). This is consistent with seedlings from invasive populations being more responsive to increased resources. Overall, the strong P response of seedlings from the invasive range together with comparable performance of seedlings without P addition suggests that seedlings from invasive populations may only have a competitive advantage in high P conditions [58].

It should be noted that this study focused on interactions with generalists in the native range since we collected soil more than 200 m away from any *T. sebifera* trees [59]. It is possible that we would have observed overall more negative effects of interactions with the soil biota had we used soil collected near conspecifics [60]. The interactions of *T. sebifera* seedlings of different origins might also differ if the soil community included more specialists [21]. If *T. sebifera* interacts with few specialists in the introduced range, the results of this study may help to understand the role of plant-soil interactions and soil resources in invasions. Research conducted on *Robinia pseudoacacia* by collecting soil from native, expanded (naturalized), and invasive ranges indicated that invasive plants are successful due to acquiring mutualisms and meanwhile, escaping from pathogens to gain a net positive effect of soil biota [11]. Further studies conducted in areas where *T. sebifera* is naturalized but not invasive [41], [61] would increase our knowledge of the role soil communities play in range expansion of *T. sebifera*.
